# The Impact of Rheology on Viscous Oil Displacement by Polymers Analyzed by Pore-Scale Network Modelling

**DOI:** 10.3390/polym13081259

**Published:** 2021-04-13

**Authors:** Iselin C. Salmo, Ken S. Sorbie, Arne Skauge

**Affiliations:** 1 Department of Chemistry, University of Bergen, N-5020 Bergen, Norway; arne.skauge@uib.no; 2 Energy Research Norway, N-5020 Bergen, Norway; k.sorbie@hw.ac.uk; 3 Institute of Petroleum Engineering, Heriot-Watt University, Edinburgh EH14 4AS, UK

**Keywords:** polymer rheology, viscous oil, pore network modelling, pore scale, enhanced oil recovery

## Abstract

Several experimental studies have shown significant improvement in heavy oil recovery with polymers displaying different types of rheology, and the effect of rheology has been shown to be important. These experimental studies have been designed to investigate why this is so by applying a constant flow rate and the same polymer effective viscosity at this injection rate. The types of rheology studied vary from Newtonian and shear thinning behavior to complex rheology involving shear thinning and thickening behavior. The core flood experiments show a significantly higher oil recovery with polyacrylamide (HPAM), which exhibits shear thinning/thickening behavior compared to biopolymers like Xanthan, which is purely shear thinning. Various reasons for these observed oil recovery results have been conjectured, but, to date, a clear explanation has not been conclusively established. In this paper, we have investigated the theoretical rationale for these results by using a dynamic pore scale network model (DPNM), which can model imbibition processes (water injection) in porous media and also polymer injection. In the DPNM, the polymer rheology can be shear thinning, shear thinning/thickening, or Newtonian (constant viscosity). Thus, the local effective viscosity in a pore within the DPNM depends on the local shear rate in that pore. The predicted results using this DPNM show that the polymer causes changes in the local flow velocity field, which, as might be expected, are different for different rheological models, and the changes in the velocity profile led to local diversion of flow. This, in turn, led to different oil recovery levels in imbibition. However, the critical result is that the DPNM modelling shows exactly the same trend as was observed in the experiments, viz. that the shear thinning/thickening polymer gave the highest oil recovery, followed by the Newtonian Case and the purely shear thinning polymer gave the lowest recover, but this latter case was still above the waterflood result. The DPNM simulations showed that the shear-thinning/thickening polymer show a stabilized frontal velocity and increased oil mobilization, as observed in the experiments. Simulations for the shear-thinning polymer show that, in high-rate bonds, the average viscosity is greatly reduced, and this causes enhanced water fingering compared to the Newtonian polymer case. No other a priori model of the two-phase fluid physics of imbibition, coupled with the polymer rheology, has achieved this degree of predictive explanation, of these experimental observations, to our knowledge.

## 1. Introduction

Polymer flooding is a mature, enhanced oil recovery (EOR) method applied to improve the mobility ratio between oil and the injected aqueous fluid, and, thus, increase volumetric sweep efficiency [1]. Water-soluble polymers are added to the injection water to increase the viscosity of the injected solution, which reduces the water-oil mobility ratio.

More recently, it has been found that polymer flooding is very efficient even for oil recovery of extra heavy viscous oil [2]. For example, polymer flooding applied in Pelican Lake showed accelerated oil production at a very adverse mobility ratio. Many other studies have investigated the application of polymer flooding for such heavy viscous oils [3,4,5,6,7,8,9,10,11,12]. These studies definitively established that, contrary to the conventional displacement theory, significant incremental oil could be produced by injecting the polymer in very viscous oils and this oil was produced in an accelerated manner. This acceleration has recently been shown to occur by viscous crossflow of oil into established water fingers [8,13,14].

Polymer solutions are non-Newtonian fluids, which means that the effective viscosity of a given polymer solution is dependent on the details of the flow conditions [15], and this is especially true in the complex flow field within a porous medium [16]. Two main types of polymers are used for EOR purposes: the synthetic polymer partially hydrolyzed polyacrylamide (HPAM) and the biopolymer xanthan. Biopolymers such as xanthan only exhibit Newtonian and shear-thinning behavior [1]. However, due to its elasticity, HPAM shows both shear thinning at lower flow rates and also shear-thickening behavior at moderate to high flow velocities in porous media [17]. We refer to this as shear thinning/thickening behavior. Since the earliest days of polymer flooding, HPAM has been, by far, the most widely used material in the polymer flooding field and pilot projects [18].

There have been several studies on the effect of polymer flooding with different rheological properties on oil recovery, both at the core scale [19,20,21,22] and in micro-model channels [23,24]. Some experimental results found lower residual oil with the polymer [25] and such results in numerical studies have attributed these findings to viscoelastic and shear thickening effects [26,27,28]. Studies in China related to polymer application in the Daqing field stated that the elastic properties of the polymer solution increased the oil recovery in porous media with no increase in the pressure gradient and that the oil recovery of HPAM is higher than that of xanthan [22]. Vermolen et al. (2014) [20] conducted polymer flooding experiments using the same in-situ viscosity and flooding rate and observed a reduction in residual oil saturation when injecting a highly viscoelastic polymer in cores with low viscosity oil. However, they did not observe a reduction when high viscosity oil or low viscosity elastic polymers were used. These workers did not include Newtonian (glycerol) flooding experiments in their study. Levitt et al. (2013) [6] found that HPAM nearly doubled the tertiary oil recovery compared to injection of xanthan and Newtonian fluid (Sucrose solution). Vik et al. (2018) [21] utilized in-situ saturation measurements in their sandstone slab flooding experiments to characterize the water/polymer oil displacements in great detail. The results showed a difference in the displacement pattern for HPAM compared to xanthan (shear thinning) and Newtonian fluids with same effective viscosity within the porous medium. 

Clark et al. (2016) [23] published a study suggesting that elastic turbulence was the main reason for the reduction in residual oil saturation for HPAM compared to xanthan, due to the generated fluctuating pressure field, which, in turn, destabilize trapped oil. De et al. (2018) [24] extended this study and found that viscoelastic polymers could displace more oil compared to Newtonian fluids and nearly inelastic shear-thinning polymers at similar capillary numbers. It is of key importance to understand the effect of polymer rheology on multiphase flow of polymers in porous media. This is both of scientific and commercial importance since HPAM is being applied in the polymer flooding of a number of fields around the world. 

The aim of the study is to explain why polymers with different rheological behavior, but the same average (or effective) viscosity within the porous medium, show large changes in oil recovery. The change in microscopic displacement efficiency has earlier been attributed to viscoelastic properties of some polymers. Here, we are addressing the impact of a shear viscosity relationship on frontal displacement and possible local fluid microscopic diversion of the injected fluids. These local flow properties need to be studied at the pore level and we have used our recent developed dynamic pore network model (DPNM) designed to handle variation in polymer rheology properties [29,30,31]. 

In more detail, we compare water (Newtonian fluid) injection with six fluids with increased viscosity, i.e., xanthan polymer solution (Newtonian/shear-thinning), glycerol (Newtonian fluid), and HPAM polymer solution (Newtonian/shear-thinning/shear-thickening). The pore network model used in this paper is a dynamic imbibition model based on the work of Li et al. (2016, 2017) [30,31] as extended by Zamani et al. (2019) [29]. “Dynamic” in this context means unsteady state modelling of the imbibition (water injection) process. This model includes both piston-like and film flow/snap-off pore scale processes and the effects of both viscous and capillary forces are included. The dynamic behavior of the network model is essential to model the effects of rheology, since it is the polymer rheology, which changes the local balance between the viscous/capillary forces that allows fluid microscopic diversion, and, hence, improved incremental recovery, to emerge. For more details and a full description of the dynamic water flood network code, we refer to the PhD thesis by Li (2016) [31], and, for the polymer implementation, we refer to the Appendix A and the paper by Zamani et al. (2019) [29].

## 2. Experimental Data Used from Literature

In this paper, we utilize experimental data from previously reported viscous oil displacement experiments (*µ_o_* ≈ 430–490 cP/*µ_o_* ≈ 430–490 mPas), which were all carried out as secondary injections in 2D sandstone slabs, viz. the displacement of viscous oil from initial water saturation, *S_wi_*, using water, xanthan polymer solution, glycerol solution, and HPAM polymer solution [21]. The experimental data is based on published results given in a paper by Vik et al. [21]. These experiments are consistent with other published data [19,20,21,22], and we have chosen these data due to the detailed description of results. The similar viscosity range and all experiments are direct injections of the polymer without any prior waterflood. The rock material used in these 2D experiments was a fairly homogenous Bentheimer sandstone with dimensions approximately 30 cm × 30 cm × 2 cm. The brine composition used to establish the initial water saturations (*S_wi_*) contained 6.0 g/kg NaCl and 1.0 g/kg NaHCO_3_, which gives a total of 7000 ppm (mg/kg) total dissolved salts. A wettability study indicated that the system was in an intermediate wettability state. Other characteristics of the experiments, which are relevant to this study, are summarized in Table 1. The polymer concentrations were adjusted so that the in-situ viscosity was comparable at the expected in-situ shear rates. The experimental setup is described in detail in the paper by Vik et al. (2018), where more information about the experiment is given [21].

**Figure 1:**
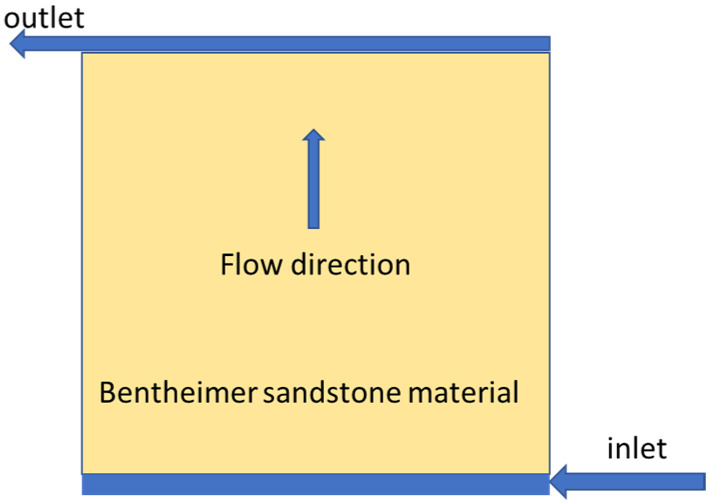
Tubing placement for horizontal inlet and outlet. The differential pressure is measured between inlet and outlet lines.

Pressure measurements were made at the inlet and outlet ends of the slab, as shown in Figure 1, with fluid (water or polymer) injection at the bottom and production at the top. The rock slabs were installed vertically in an X-ray scanner. Iododecane was added to the oil to achieve contrast for the x-ray imaging. The x-ray images gave detailed information on the dynamics during the displacement.

**Figure 2:**
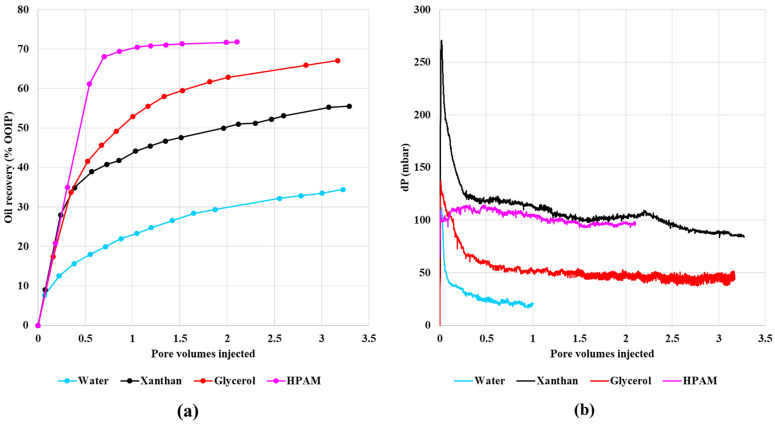
(**a**) Oil recovery and (**b**) differential pressure during secondary injections at an injection rate of 0.05 mL/min as a function of pore volumes. Based on data from Reference [21].

The experimental results of Vik et al. (2018) [21] for oil recovery and differential pressure as a function of pore volumes (PV) injected is presented in Figure 2a,b, respectively. The oil recovery is significantly increased when compared with the waterflood by the more viscous (polymer or glycerol) injection fluid. Water flooding gives the lowest recovery, which is followed by the shear-thinning polymer xanthan, and then the Newtonian glycerol solution, with the shear thinning/thickening polymer HPAM yielding the highest oil recovery. Thus, a significant difference in oil mobilization and recovery is observed experimentally depending on the fluid rheology. The experimental flow rate was set at 0.05 mL/min, which was achieved by imposing a rapid but gradual build-up to this final rate. The gradual buildup of flow rate counteracts the quick decline in differential pressure seen in all other experiments. The total differential pressures near the endpoint stabilized oil production do not display the same trend and cannot explain the large differences in observed oil production. The rheological properties of the water-soluble polymers are well documented in Sorbie [1], where Xanthan shows shear thinning, while HPAM display shear thinning at a low shear rate, which changes to shear thickening at a high shear rate.

X-ray images were captured during secondary injections for the experiments conducted by Vik et al. (2018) [21]. The X-ray images showed a clear difference in the displacement process for water, Xanthan, glycerol and HPAM. The most extreme immiscible fingering of water into the oil was observed for the waterflood leading to the early breakthrough of water and less efficient oil displacement. The displacement stability was significantly different for the different and more viscous injection fluids. HPAM, with its shear-thinning and thickening behavior, showed the most stable front and most efficient production. The Xanthan resulted in a lower recovery compared to glycerol due to unswept oil between the fingers or channels.

## 3. Dynamic Pore Network Model (DPNM) Simulation

The parameters used in the DPNM simulations are given in Table 2. The pore network itself is a 2D model, with injection from the left to the right. We initially show the 2D results due to computational time, storage, and better visualization. However, very similar results are observed in 3D [32]. The oil viscosity simulated was taken as the mean of the experimental oil viscosities (Table 1), i.e., *µ_o_* = 466 cP. All DPNM simulations were run for one pore volume as all simulations had reached a steady state after 1PV of injection. Thus, there is no need to inject more fluid.

**Figure 3:**
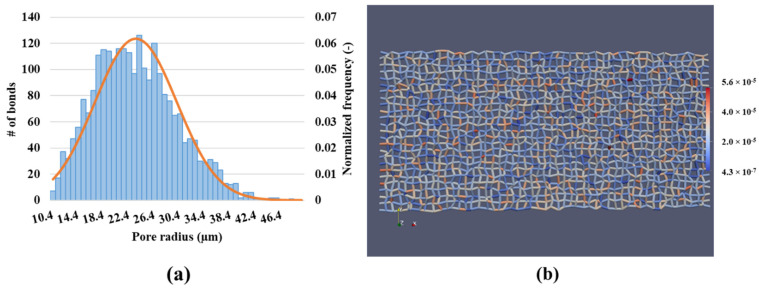
(**a**) pore size distribution, truncated normal distribution, and (**b**) pore network model used in this study.

Figure 3a shows the pore size distribution, together with the normal distribution of pore sizes. The minimum and maximum pore size is 10 µm and 50 µm, respectively. The pore network model is visualized in Figure 3b.

The idea behind the experimental studies of Vermolen et al. (2014) [20], and also in the DPNM numerical experiments presented here, was to use the same in-situ apparent polymer viscosity and flooding rate for all flooding experiments. The rheological properties for the DPNM simulations were chosen so that, at the chosen injection rate, the same apparent viscosity was obtained in the 2D network in single-phase conditions (i.e., at *S_w_* = 1) for each of the model rheologies of the polymers (see below). The apparent viscosity of the in-situ (polymeric) fluid, $\mu_{app}$, was calculated by the formula in Equation (1).

(1) $$\mu_{app}=\frac{\Delta P_{p}}{\Delta P_{w}}\mu_{w}$$

where ΔP is the pressure drop across the model, where subscript p is polymer and w is water. $\;\mu_{w}$ is the viscosity of water ($\mu_{w}$ = 1 cP in all calculations in this paper).

**Figure 4:**
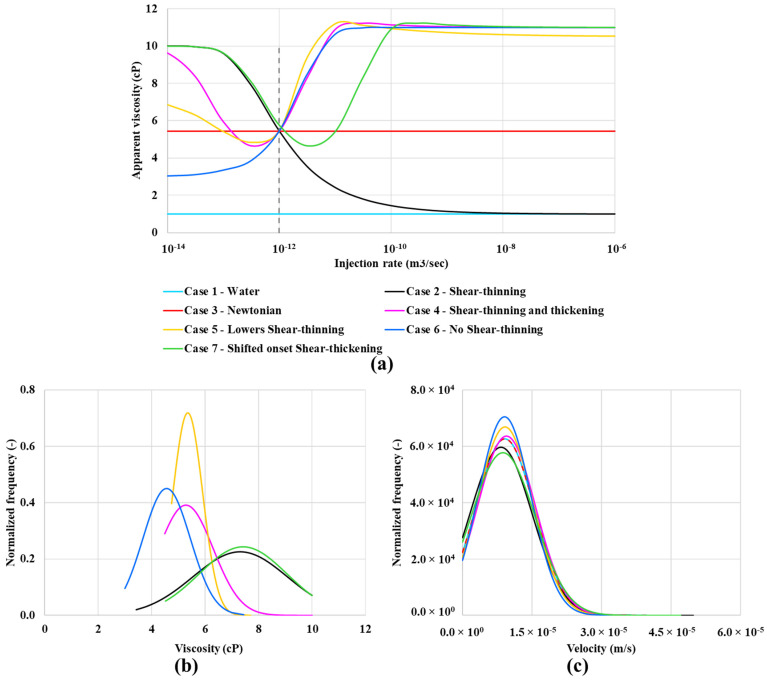
(**a**) in-situ rheology of water and six “polymer” solutions, (**b**) viscosity distribution at an injection rate of 1 × 10^−12^ m^3^/s and (**c**) corresponding velocity distribution, all in a single phase flow.

Six types of polymer solutions were simulated in our numerical DPNM study: (i) shear-thinning, (ii) Newtonian only, (iii) combined shear-thinning and thickening, (iv) combined shear-thinning and thickening with reduced shear-thinning effect, (v) shear-thickening only, and (vi) combined shear-thinning and thickening with a shifted onset of shear-thickening. These are identified by the case number for clarity in the analysis of results below. However, we note that all fluids are Newtonian at sufficiently low flow rates. The single phase (*S_w_* = 1) in-situ rheology curves for these six cases are shown in Figure 4a. All cases have approximately the same average apparent viscosity at the reference injection rate (Q = 1 × 10^−12^ m^3^/s) in the porous medium. This value is ~ 5.5 cP, as seen from the crossing point in Figure 4a. However, in a particular pore, the fluid may have a local viscosity different from the average network value, according to the particular rheological model of that case. Figure 4b,c show the distribution functions for the local viscosity and the local velocity at the pore scale, respectively. 

Throughout this paper, we will refer to the water case and these six “polymer” cases as:

- Case 1—Water flood,
- Case 2—Shear-thinning only (no shear thickening region),
- Case 3—Newtonian “polymer” with a fixed viscosity (~ 5.5 cP in this Case) equivalent to the in-situ effective viscosity of the other polymers at the base Case flow rate (Q = 1 × 10^−12^ m^3^/s),
- Case 4—Shear-thinning and thickening polymer (most like an HPAM),
- Case 5—Lower shear-thinning, like case 4 but a lower Newtonian plateau at a low shear rate but the same shear-thickening region as case 4,
- Case 6—No shear-thinning—case with a lower viscosity Newtonian plateau going into the shear-thickening region without showing any shear-thinning,
- Case 7—Shifted onset shear-thickening—the same curve as Case 4 (shear-thinning/thickening) but moved to the right, as shown in Figure 4a.

Case 5—lower shear-thinning polymer shows the highest peak in viscosity distribution at around 5.5 cP, as shown in Figure 4b, at approximately the same viscosity as case 4—combined shear-thinning and thickening polymer. Case 2—shear-thinning and Case 7—shifted onset of shear-thickening polymers show similar viscosity distribution in a single-phase flow. The velocity distribution functions in Figure 4c indicate that all polymer cases have a very similar velocity distribution.

**Figure 5:**
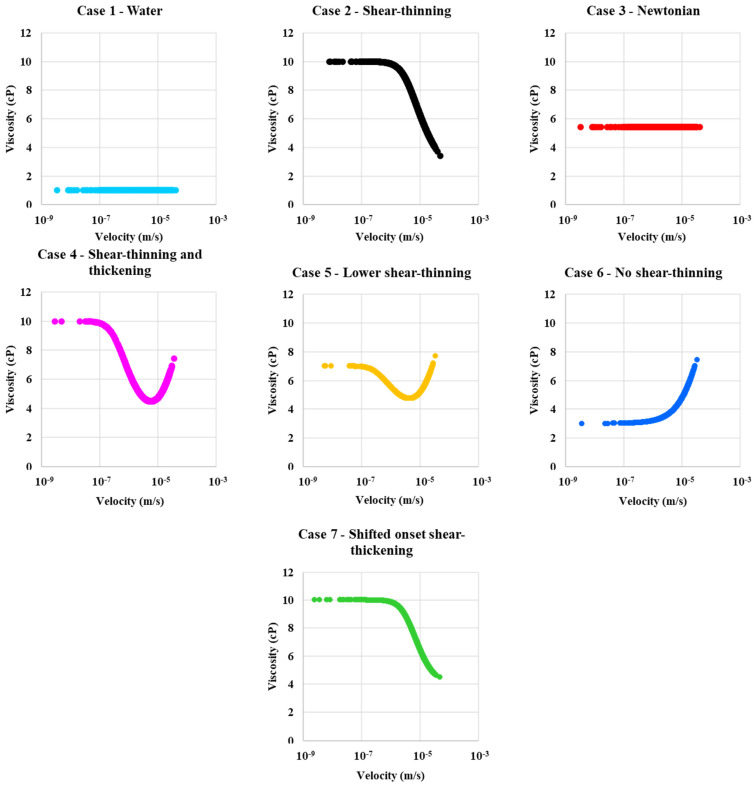
In-situ viscosity as a function of velocity for Case 1—water, Case 2—shear-thinning, Case 3—Newtonian, Case 4—shear-thinning and thickening, Case 5—lower shear-thinning, Case 6—no shear-thinning, and Case 7—shifted onset of a shear-thickening polymer.

Figure 5 shows the calculated in-situ viscosity vs. fluid velocity for case 1—water flood and the six polymer cases. Case 1—water and Case 3—Newtonian in-situ viscosities are constant, as expected, as shown in Figure 5. The other polymer in-situ rheograms in Figure 5 have the same shapes as the apparent viscosity curves in Figure 4a.

## 4. Results and Discussion

### 4.1. Base Case Dynamic Network Simulations

The DPNM simulation have been performed to model water and polymer displacement of oil in a 2D network model of the porous medium. The purpose of the simulations is to understand the impact different rheology values have on oil mobilization. The central objective is to understand the reason for the experimental observations by reproducing a similar qualitative pattern of the results using a dynamic network model. If our DPNM achieves this objective, then our explanation of these experiments qualified as a plausible explanation. It is not a “proof”, but, at present, there is no other model which is currently qualified even to this level.

**Figure 6:**
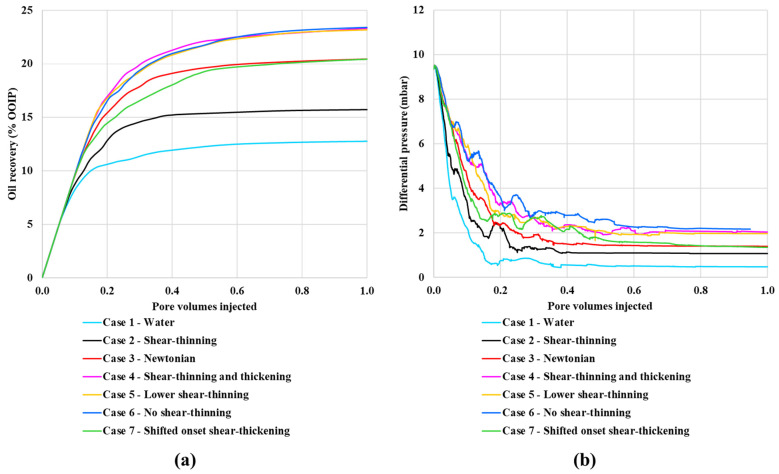
(**a**) Oil recovery and (**b**) differential pressure for pore network modelling with an injection rate of 1 × 10^−12^ m^3^/s as a function of pore volume injected. The differential pressure is the difference between inlet and outlet pressure of the network model, analogous to the experimental data.

The simulations are anchored on experimental data for displacement of a viscous oil (*μ_o_* = 466 cP). The results for oil recovery and differential pressure as functions of pore volumes injected are presented in Figure 6. Figure 6a shows oil recovery vs. PV injected and Figure 6b shows dP vs. PV. The simulation predictions clearly agree very well qualitatively with the experimental results in Figure 2, when we compare case 1–4 with the experimental results (Figure 2). To our knowledge, this result is novel in that no other model has achieved even a qualitative explanation of these experimental findings. Furthermore, since we have access to every aspect of the model, we are able to analyze exactly why we reproduce exactly the same DPNM predictions, as observed experimentally. Our analysis (presented below) shows that changes in local pore velocity due to the various rheology relationships leads to different levels of local (pore-level) microscopic fluid diversion which, in turn, impact oil recovery.

In addition, we have included cases 5–7 with slightly different rheologies compared to case 2–4, which investigate how these polymers impact the recovery and fluid flow field. The results in Figure 6a show that the final oil recovery increases in the order: water flood < shear-thinning (xanthan like) polymer < Newtonian (glycerol like) = shifted onset of shear-thickening polymer < shear-thinning/thickening (HPAM like) = lower shear-thinning = no shear-thinning polymer (i.e., Case 1 < Case 2 < Case 3 = Case 7 < Case 4 = Case 5 = Case 6). These results show the same order as the experimental results in Figure 2.

### 4.2. Fluid Displacement Patterns

**Figure 7:**
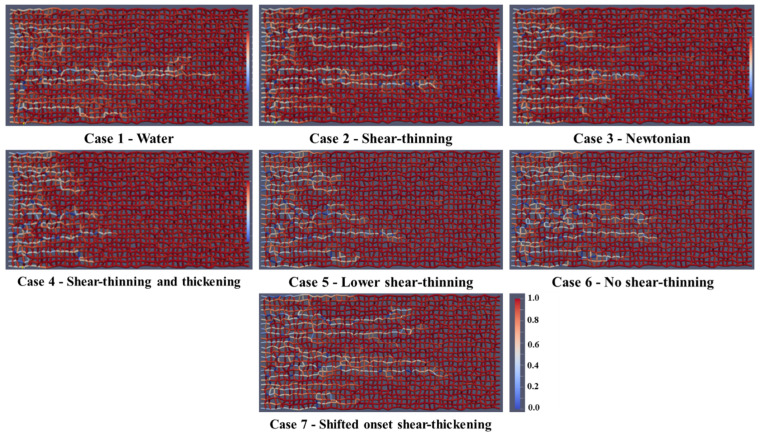
Fluid distribution (oil and water) after 0.1 PV of injected fluid.

Figure 7 shows the fluid distribution of oil (red) and water (white) after 0.1 PV of injected fluid for Case 1—water, Case 2—shear-thinning, Case 3—Newtonian polymer, Case 4—shear-thinning/thickening, Case 5—lower shear-thinning, Case 6—no shear-thinning, and Case 7—shifted onset of shear-thickening injection (from left to right). The injection fluid front has progressed further toward the production well for water flooding (Case 1) and the shear-thinning case (Case 2) because of the viscous instability leading to mainly one dominating channel. In the shear-thinning and thickening (Case 4), lower shear-thinning (Case 5), and no shear-thinning cases (Case 6), the front appears more stable, with several active flow channels, thus, sweeping the oil from the network in a more efficient manner.

**Figure 8:**
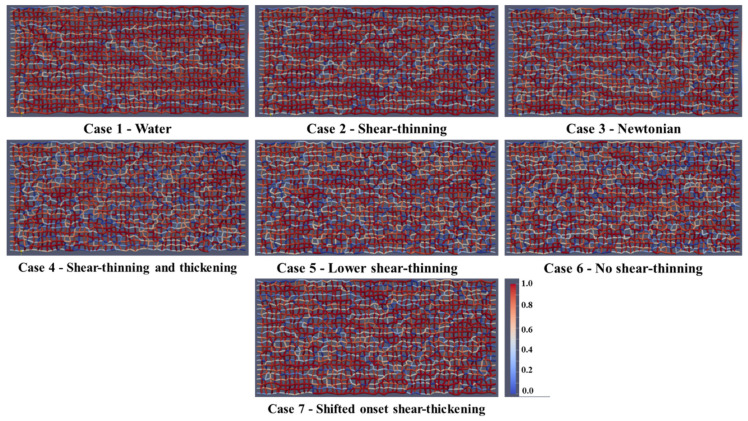
Fluid distribution (oil and water) after 1.0 PV of injected fluid.

The corresponding fluid distributions for the same 7 cases above are shown after 1 PV of injected solution in Figure 8. As the total recovery is low, not much of the oil is displaced. However, the more complex polymer (showing shear-thickening behavior—cases 4 to 6) displaces more than the other polymer cases and water. There is a more pronounced fingering pattern in the water (Case 1) and shear-thinning (Case 2) fluid injections and more bypassed oil, while the displacement process in the HPAM case (Case 4) is visibly and significantly more efficient.

### 4.3. In-Situ Viscosity Distribution

**Figure 9:**
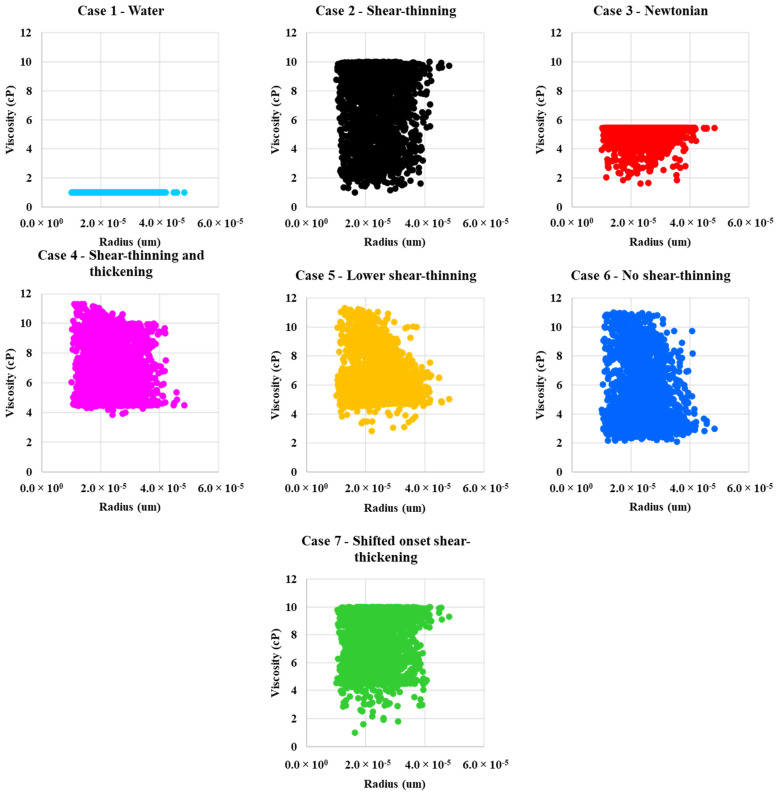
In-situ viscosity as a function of radius for case 1—water, case 2—shear-thinning, case 3—Newtonian, case 4—shear-thinning and thickening, case 5—lower shear-thinning, case 6—no shear-thinning, and case 7—shifted onset of shear-thickening polymer.

**Figure 10:**
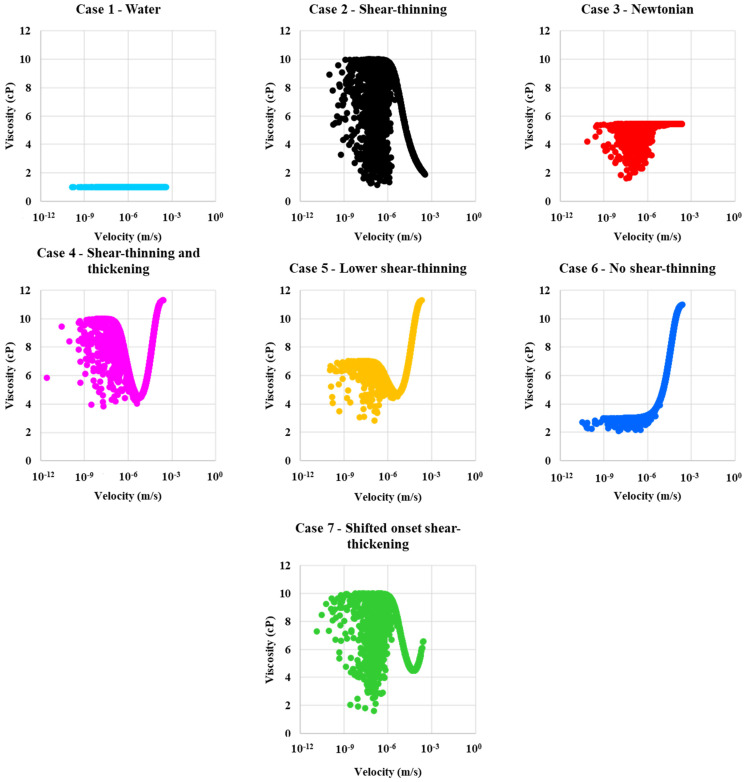
In-situ viscosity as a function of velocity for case 1—water, case 2—shear-thinning, case 3—Newtonian, case 4—shear-thinning and thickening, case 5—lower shear-thinning, case 6—no shear-thinning, and case 7—shifted onset of a shear-thickening polymer.

Figure 9 and Figure 10 show the in-situ viscosity distribution for the two-phase (water or polymer solution → oil) displacements as a function of pore radius and as a function of the local velocity in the network bonds, respectively. Case 1—water has constant viscosity at all radii (*μ*_w_ = 1 cP). All cases, except the water (Case 1), are influenced by dual occupancy of a non-Newtonian polymer and Newtonian water in the network bonds. Case 3—Newtonian polymer has viscosity of 5.44 cP. However, in some bonds, it is lower due to miscible mixing of the “polymer” with water present in water films. The mixing occurs more in bonds with a smaller pore radii. Case 2—shear-thinning and case 6—no shear-thinning polymers experiences both high and low viscosity in narrow and wide pores, and they, therefore, show a wide distribution in viscosity. Case 4—shear-thinning and thickening, case 6—lower shear-thinning, and case 7—shifted onset of shear-thickening polymer experience a wide range of viscosities across all pore radius values. However, they appear to rarely go below a viscosity of approximately 4 cP.

Figure 10 shows the corresponding viscosity distribution for each of the above cases as a function of the local velocity in the network bonds for the two-phase displacements. The velocity field for water injection (Case 1) (which has a constant *μ*_w_ = 1 cP) show constant viscosity for all velocities, as expected for Newtonian fluids. All polymers are influenced by mixing with resident water in the bonds, like in Figure 9. The mixing with water appears at lower local velocities, and, if we combine with the information for Figure 9, mixing also appear for smaller pore radii. The in-situ viscosity in these two-phase displacements as a function of velocity is, when we exclude the mixing effect, broadly similar in shape as the single-phase cases in Figure 5.

**Figure 11:**
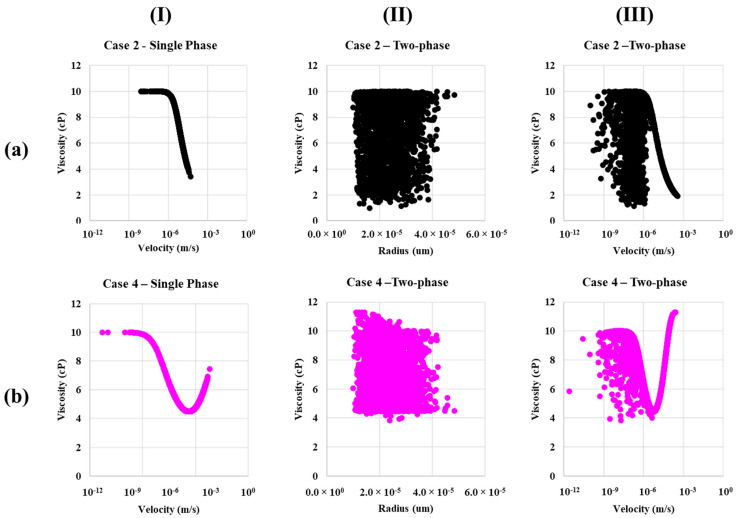
Single-phase viscosity versus velocity and multiphase viscosity versus radius and viscosity versus velocity for (**a**) case 2—Shear-thinning and (**b**) case 4—Shear-thinning and thickening.

A comparison of shear-thinning (Case 2—Xanthan like) and shear-thinning and thickening (Case 4—HPAM like) is included in Figure 11. The in-situ viscosity is compared both for a single-phase flow and two-phase flow situation. Single phase data (I) show there is a larger span in viscosity function of bond velocity for case 2 as the shear thinning only extends to lower viscosity, while case 4 has a minimum viscosity (4.3 cP) at the apparent Newtonian plateau. The two-phase viscosity for variation in bond radius (II) has a larger variance for shear-thinning due to water-polymer mixing in two phase flow and the span in viscosity seen in a single phase. Furthermore, these factors explain the variance in viscosity for the velocity plot (III). In summary, shear-thinning (Case 2) has a larger variance in viscosity and has deceasing viscosity at the largest velocity, while shear-thinning and thickening (Case 4) show increases for the largest bond velocities.

The velocity profile in Figure 10 and Figure 11 showing higher viscosity for case 4 (shear-thinning and thickening) compared to case 2 (shear-thinning) and the fluid displacement pattern in Figure 7 and Figure 8 showing more bypassed oil and more of a finger pattern for case 2 than case 4, which helps to explain why there is more fluid diversion and, thus, more efficient oil displacement for the case 4 polymer. 

### 4.4. In-Situ Velocity Distributions

**Figure 12:**
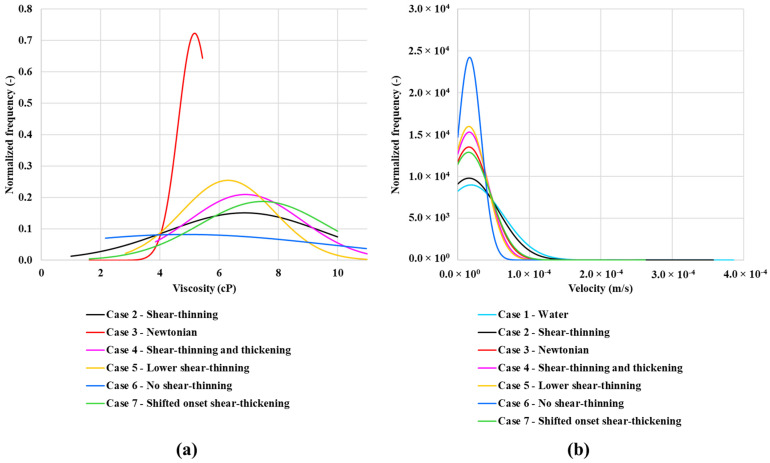
Normalized frequency for in-situ (**a**) viscosity and (**b**) velocity.

The in-situ velocity and viscosity distributions presented above can help to explain the order of oil recovery. As shown in Figure 12b, all of the normalized frequency curves for water and polymer peak at approximately the same velocity. The lower the velocity frequency, the broader is the velocity distribution. This result in lower viscosities for case 2—shear-thinning polymer (see Figure 12a) as it goes towards water viscosity at higher rates (Figure 4a). A similar trend is observed for case 7—shifted onset of shear-thickening polymer. However, this has a higher frequency of higher viscosities, resulting in higher oil recovery. The viscosity distribution is different from the single-phase viscosity distribution in Figure 4b, showing both a lower frequency and wider distribution. Hence, the velocity (and, therefore, the viscosity) field changes going from a single-phase flow to a multi-phase flow.

### 4.5. Pore Occupancy Statistics—Base Case Simulations

**Figure 13:**
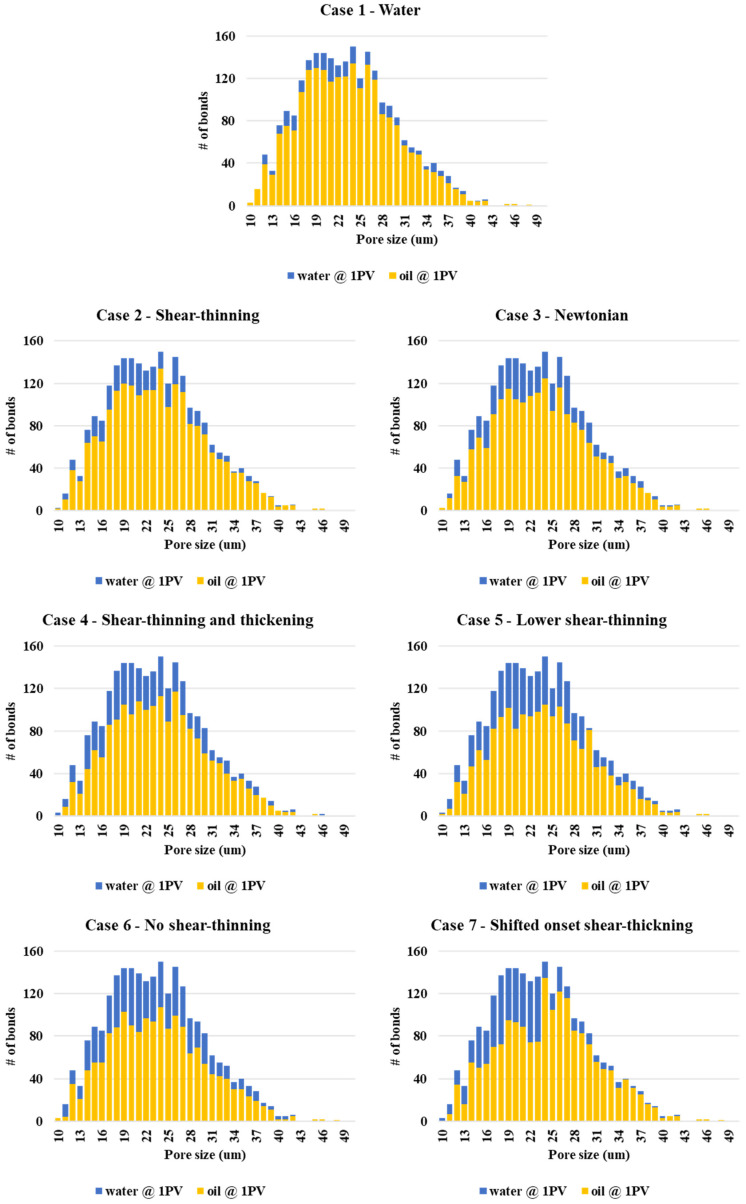
Polymer and water pore (bond) occupancy.

The pore occupancy statistics of the water and oil phases at the end of simulation are given in Figure 13. In this figure, the overall histogram is the frequency distribution of “pores” of a given size interval, as shown in Figure 3a. The final fractional phase occupancy of oil and water for each pore size are shown in yellow and blue, respectively. There is 50% filling criteria that implies that the additional water saturation of the pore is ≥ 50%. This is required since the dynamic imbibition model of Li et al. (2017) [30] can have both oil and water in both bulk and films, and this criterion registers that significant water diversion into these pores has occurred. The phase occupancy of water and oil is shown at the end of the waterflood (Case 1) at the top of Figure 13. The relatively small amount of water occupancy is because of the low recovery in the 2D network. However, it is the comparison of the other network simulations relative to the water flood case that is relevant here. These simulations represent the different rheological models for the “polymer” and all the cases described above are shown in Figure 13. The main point to note from these simulations is that the Newtonian (Case 3) and purely shear-thinning polymer (Case 2) certainly improve upon the waterflood, but it is the cases which have a shear thickening region in their rheological mode that divert more water into the mainly intermediate-sized pores in the network, thus, improving the oil recovery in accord with the results shown above in Figure 6. The importance is that the order of improved recovery from the highest to lowest is case 4—shear thinning-thickening (and other models with a shear thickening region—case 5, 6, and 7), which is followed by the Newtonian polymer (Case 3), then the shear-thinning (Case 2), and then followed by the waterflood (Case 1). This is the same as the order observed experimentally.

### 4.6. Lower Flow Rate Results—Recoveries

In the base case simulations presented above, the rheological functions shown in Figure 4a were chosen, such that they gave the same effective viscosity at a given fixed flow rate (Q = 1 × 10^−12^ m^3^/s). A set of simulations was carried out for three of these rheological models at a lower flow rate, Q = 1 × 10^−13^ m^3^/s; viz. Case 3—Newtonian polymer, case 4—shear-thinning and thickening polymer, and case 6—no shear-thinning polymer (but with a shear thickening region). The rheology curves for these three polymers are still the same as previously (Figure 4a). Thus, they do not have the same apparent viscosity at the given injection rate. 

**Figure 14:**
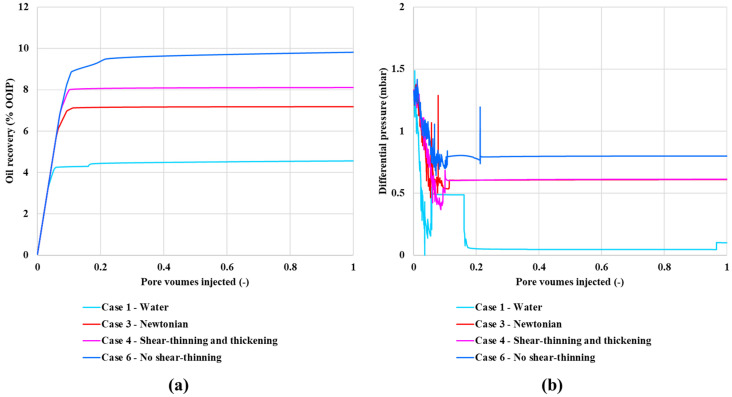
(**a**) Oil recovery and (**b**) differential pressure for pore network modelling with an injection rate of 1 × 10^−13^ m^3^/s as a function of pore volumes.

**Figure 15:**
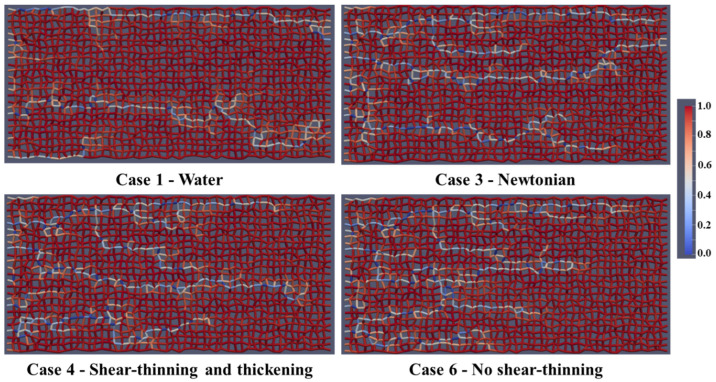
Fluid distribution (oil and water) after 0.1 PV of injected fluid with Q = 1 × 10^−13^ m^3^/s.

Figure 14 shows the oil recovery and the differential pressure as a function of pore volumes for the three polymer cases, compared with the corresponding low rate waterflood. Note that the oil recoveries are even smaller than the base case since capillary forces are relatively stronger and make it more difficult for viscous forces to displace the oil. In these low rates and dynamic simulations, the oil recovery increases in the order Waterflood < Newtonian < shear-thinning and thickening < the no shear-thinning (i.e., Case 1 < Case 3 < Case 4 < Case 6). In the base case (Q = 1 × 10^−12^ m^3^/s), the combined shear-thinning and thickening (Case 4) and no shear-thinning (Case 6) resulted in approximately the same oil recovery, but, at this lower rate, the case 6—no shear-thinning (but with a thickening region) gives higher oil recovery and a higher differential pressure. It is not clear why this is the case. It is counter-intuitive since we are lowering the flow rate and, at this lower flow rate, the no shear-thickening case (Case 6) has a lower Newtonian viscosity. This situation might be expected to result in a lower oil recovery. However, it appears that the oil phase displacements by the “polymer”—the degree of fluid diversion relative to the waterflood—is governed by a complex coupled interaction between the rheology of the fluid and the local balance of the viscous/capillary forces in the imbibition process. It is shown below in the fluid displacement patterns (Figure 15) that, for the no shear thinning case (Case 6), then more diversion and finger suppression occurs due to this coupled interaction.

### 4.7. Lower Flow Rate Results—Fluid Distributions

Figure 15 shows the fluid distribution of oil (red) and water (blue) after 0.1 PV polymer injected for the Case 1—water flood, Case 3—Newtonian, Case 4—shear-thinning and thickening, and Case 6—no shear-thinning cases for the lower flow rate, Q = 1 × 10^−13^ m^3^/s. The displacement front has progressed further in the Newtonian polymer case (Case 3), and the shear thinning-thickening case (Case 4) suppresses this fingering to some extent. However, the no shear-thinning case (Case 6) has a more stable front than either of the other two cases, which leads to more efficient oil displacement. 

**Figure 16:**
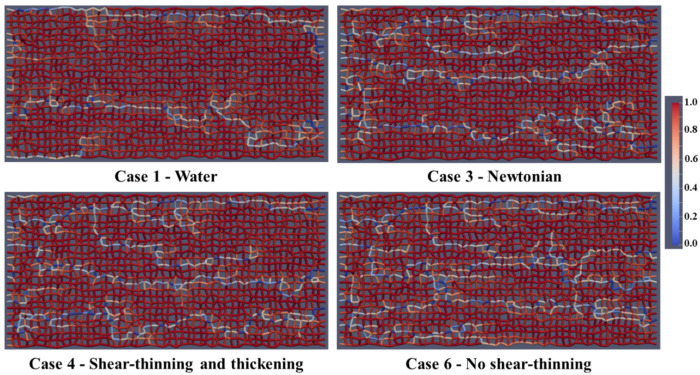
Fluid distribution (oil and water) after 1 PV of injected fluid with Q = 1 × 10^−13^ m^3^/s.

The fluid distribution after 1 PV for waterflood and the three polymer cases with an injection rate of Q = 1 × 10^−13^ m^3^/s is given in Figure 16. Very little oil has been displaced. However, there is an observable difference in fluid distribution, especially between the Case 3—Newtonian and Case 6—no shear-thinning polymer, with the Newtonian polymer (Case 3) leaving more oil behind.

### 4.8. Lower Flow Rate Results—Fluid and Velocity/Viscosity Distributions

**Figure 17:**
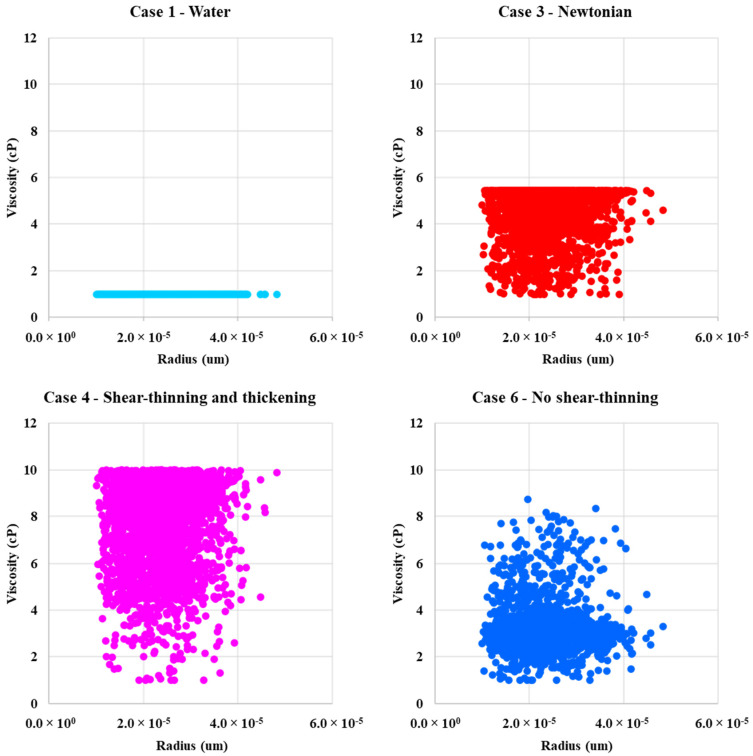
In-situ viscosity as a function of radius for Case 1—water, Case 3—Newtonian, Case 4—shear-thinning, and Case 6—thickening and no shear-thinning.

The in-situ viscosity as a function of pore radius is given, for waterflood and the three polymer cases run at the lower flow rate (Q = 1 × 10^−13^ m^3^/s) in Figure 17. The Newtonian polymer (Case 3) shows some pore level viscosity values lower than 5.44 cP, due to the mixing with water films. However, the viscosity never increased above 5.44 cP (at a full input of polymer concentration). The shear-thinning and thickening polymer (Case 4) experiences higher viscosities in the same pores as the no shear-thinning polymer (Case 6), which has a higher frequency of lower viscosities in all pores. 

**Figure 18:**
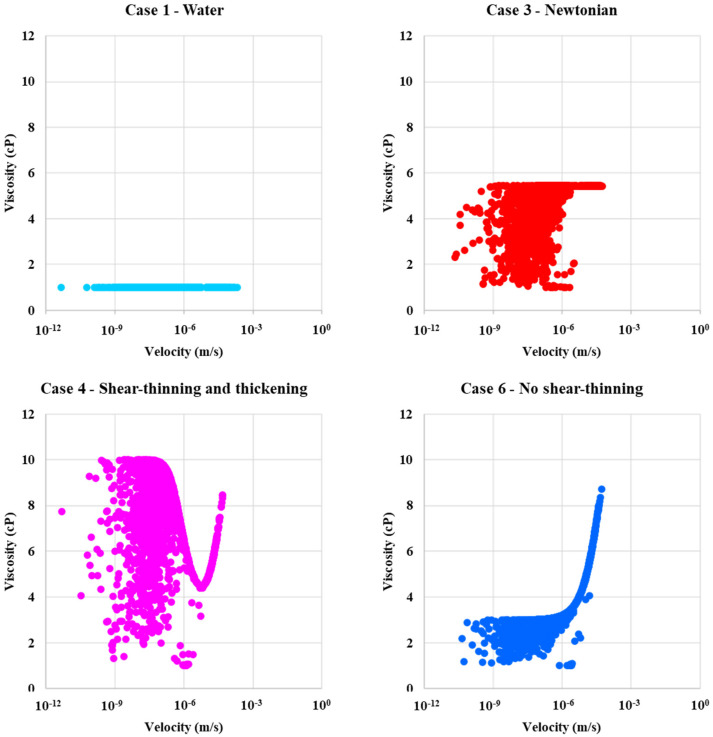
In-situ viscosity as a function of velocity for Case 1—water, Case 3—Newtonian, Case 4—shear-thinning, and Case 6—thickening and no shear-thinning.

Figure 18 shows the pore-by-pore viscosity as a function of velocity for water and the three polymer cases, and these are similar in shape to the rheology curves in Figure 4a. Compared to the viscosity versus velocity for Q = 1 × 10^−12^ m^3^/s in Figure 10, both the combined shear-thinning and thickening (Case 4) and no shear-thinning polymers (Case 6) do not reach the higher Newtonian plateau at higher velocities. From the viscosity versus velocity plots, it would be expected that the shear-thinning and thickening polymer (Case 4) increased recovery the most, due to higher viscosities. We would normally expect that the higher viscosities in Case 4—shear thinning-thickening would give a more efficient displacement, which is not the case here. We believe that this is because of the complex interaction between the flow field and the oil displacement referred to above. It also appears that the span of the viscosity distribution influences local flow diversion. This may occur because the polymer in the pores where it is shear thickening, in turn, diverts the aqueous fluid to displace oil in other (possibly adjacent) pores. However, if this direct displacement is by a low shear viscous polymer, then it will be efficient, but the local (higher low shear) viscosity will slow the direct displacement (it is a dynamic pore network model). In Case 6—no shear thinning case (but with shear thickening), the diverted lower pore flow rate aqueous phase, which is displacing oil, will have a lower viscosity. This may allow the displacement to occur more rapidly. This analysis is currently our conjectured explanation for these observations and we are currently investigating this complex coupled mechanism in more detail.

**Figure 19:**
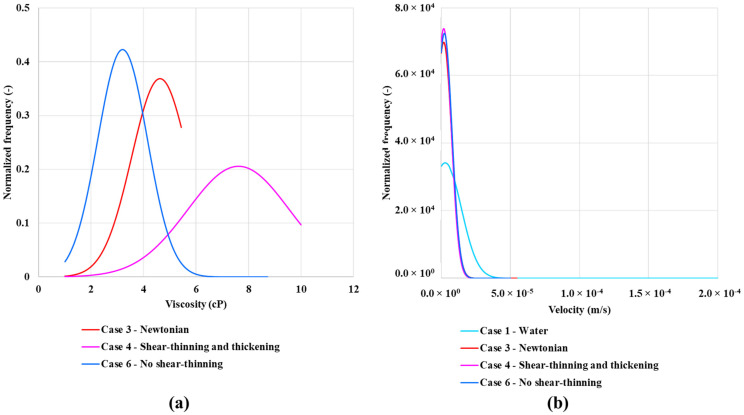
Normalized frequency for in-situ (**a**) viscosity and (**b**) velocity for Q = 1 × 10^−13^ m^3^/s.

The Case 6—no shear-thinning polymer peaks at around 3 cP and has a quite narrow distribution in viscosity (see Figure 19a). The case 3—Newtonian polymer peaks a little over 4 cP, while the case 4—combined shear-thinning and thickening polymer peaks at just under 8 cP. However, the peak is much lower compared to the two other polymers and it also has a wider distribution. The normalized velocity distribution is similar for all polymer cases. However, case 3—Newtonian and case 6—no shear-thinning polymer has a slightly wider distribution than case 4—shear-thinning and thickening polymer (see Figure 19b). The velocity distribution for case 1—waterflood is much broader and the peak in frequency is much higher than the polymer cases.

### 4.9. Lower Flow Rate Results—Phase Occupancy Statistics

**Figure 20:**
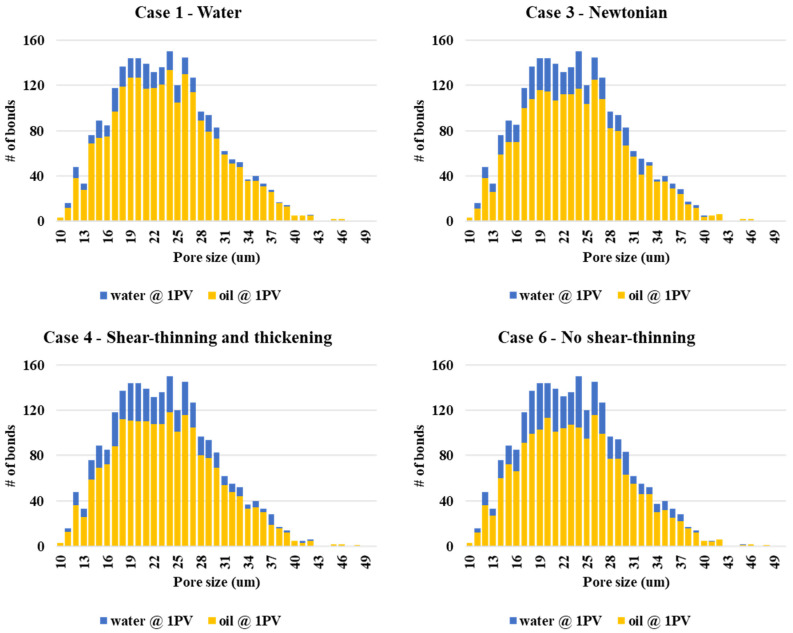
Polymer and water pore (bond) occupancy for the waterflood and the three polymers with an injection rate of 1 × 10^−13^ m^3^/s.

The pore occupancy of water and oil at the end of the simulation at the lower flow rate (Q = 1 × 10^−13^ m^3^/s) for water and the three polymer cases is given in Figure 20. Due to the lower recoveries at this flow rate, 20% filling criteria is applied for considering water entering a pore. Case 6—no shear-thinning polymer fills more bonds with the polymer (water) than case 4—shear-thinning and thickening and case 3—Newtonian polymer, as expected from the oil recovery results in Figure 14a. 

### 4.10. Longer Model Results

**Figure 21:**
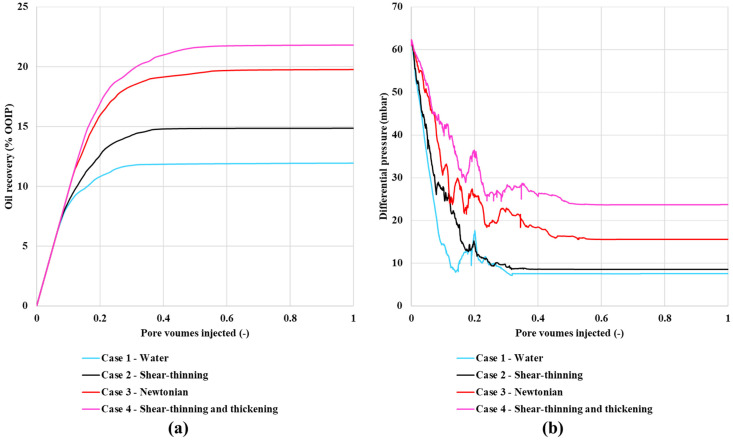
(**a**) Oil recovery and (**b**) differential pressure versus pore volumes injected for the longer (200 × 25) pore network model.

PNM simulations were also carried out on a longer model with dimensions of 200 × 25 × 1 and the oil recovery and differential pressure results are shown in Figure 21. The oil recovery is in exact agreement with the smaller base case model both for oil recovery values and the order of oil recovery, Case 1—Water < Case 2—Shear-thinning < Case 3—Newtonian < Case 4—Shear-thinning and thickening polymer.

**Figure 22:**
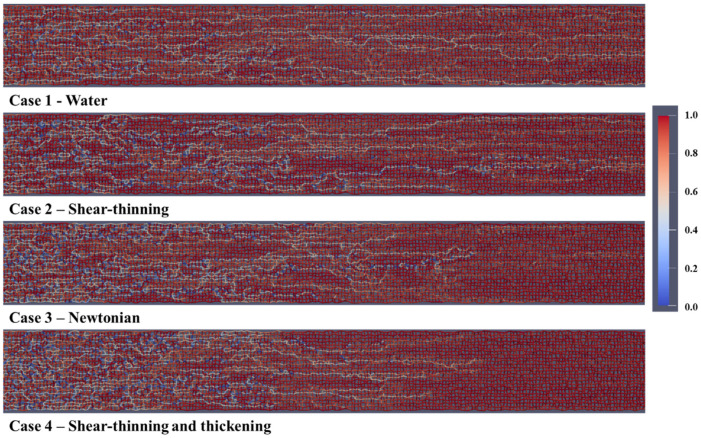
Fluid distribution (oil and water) after 0.1 PV of injected fluid with Q = 1 × 10^–13^ m^3^/s for the longer (200 × 25) pore network model.

Figure 22 shows the fluid distribution of oil (red) and water (white) after 0.1 PV of injected fluid for case 1—water, case 2—shear-thinning, case 3—Newtonian polymer, and xase 4—shear-thinning/thickening polymer injection (from top to bottom, respectively). The injection fluid front has progressed further toward the production well for water flooding (Case 1) and shear-thinning (Case 2) polymer because of the viscous instability (in agreement with the fluid distribution in Figure 7). In the shear-thinning and thickening (Case 4), the front appears more stable, with several active flow channels, thus, sweeping the oil from the network in a more efficient manner.

**Figure 23:**
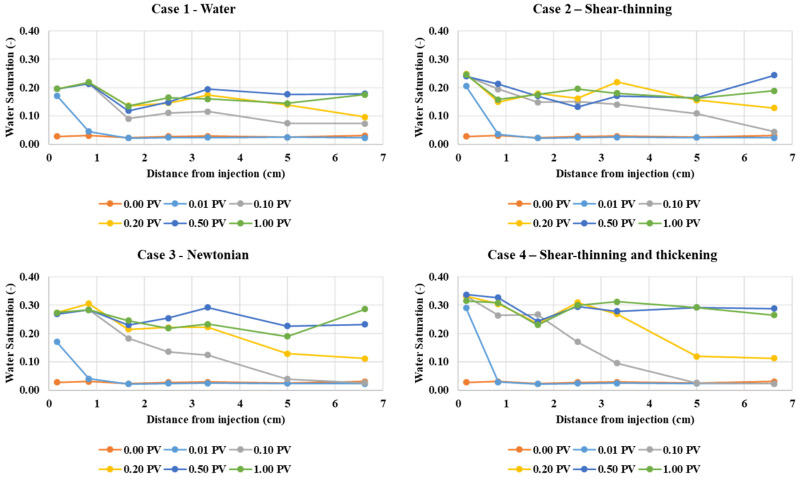
Water saturation as a function of distance from injection at different pore volumes injected for the longer (200 × 25) pore network model.

Figure 23 shows the water saturation as a function of distance from the injection at different pore volumes injected for case 1—water, case 2—shear-thinning, case 3—Newtonian, and case 4—shear-thinning and thickening. These plots confirm the observations in Figure 22 with case 4 (Shear-thinning and thickening) showing the development of a more stable water “front.” This case 4 displacement is clearly more efficient, especially compared to water (Case 1) and the shear-thinning (Case 2) polymer. The Newtonian polymer (Case 3) also exhibits a more stable frontal displacement than cases 1 and 2. This is shown in Figure 23 at 0.1 PV (grey) and 0.2 PV (yellow) for case 3. All the frontal displacement results in Figure 23 agree with the fluid distribution observations in Figure 22.

## 5. Summary and Conclusions

This paper presents a clear and consistent theoretical explanation of why polymers with different shear rheology, but having the same effective viscosity within the porous medium at a constant injection rate, can lead to very significant changes in the oil recovery efficiency. This was achieved by applying a dynamic pore network model (DPNM) of imbibition, which allowed us to implement any model for the aqueous phase rheology of the applied polymer solution. We consider that the aqueous polymer can be purely shear-thinning (like xanthan), shear-thinning, and thickening (like HPAM) or Newtonian (like glycerol). Viscous oil displacement behavior for these polymer types has been modelled in a 2D DPNM and compared with the corresponding waterflood case. It is demonstrated that our DPNM simulations give a prediction that agrees in the order of recovery with the experiment. 

The main specific findings/conclusions from this work are as follows.

- In the dynamic viscous oil network displacement simulations, all polymers (shear-thinning, shear-thinning/thickening, and Newtonian) gave improvements in oil recovery compared with the (lower viscosity) waterflood. The viscosification of the polymer was adjusted in the model until they had the same in-situ effective viscosity (at *S_w_* =1). Simulations predicted that the oil recovery performance was highest for the more complex polymer solutions including both shear-thinning/thickening behavior (i.e., HPAM-like). This, in turn, was predicted to better than the Newtonian (glycerol) “polymer”, and this was predicted to be better than for the purely shear-thinning polymer (i.e., xanthan-like polymer). This is in exact agreement with the literature experimental observations.
- The reason for this behavior in the DPNM simulations has been established by performing a detailed analysis of the flow field and its effect on the local pore viscosities for the various rheologies. The pore network model shows that the shear-thinning polymer experience higher flow velocity. Thus, lower viscosity approaches water viscosity at higher flow rates. As a result, a more severe and inefficient finger pattern is observed for a shear-thinning polymer (xanthan) compared to combined shear-thinning and thickening, in our DPNM simulations.
- In the most efficient oil recovery case for the shear-thinning/thickening (HPAM like) polymer, it was shown that this rheology results in more pore-scale fluid diversion, leading to more stable fluid displacement fronts and more efficient oil displacement.

The efficient oil recovery mechanism in the DPNM calculations by improved pore-scale diversion is demonstrated using pore occupancy statistics. Examining the pore scale occupancies, it is evident that the best performing shear-thinning/thickening (HPAM like) case causes a more injected phase fluid diversion at the local scale displacing more oil from the intermediate-sized pores (for the case studied here).

## Figures and Tables

**Table 1:** Summary of parameters for secondary flood experiments.

	Unit	Water	Xanthan	Glycerol	HPAM
**Viscous Behavior**	-	Newtonian	Shear-thinning	Newtonian	Shear-thinning and thickening
**Dimensions**	cm × cm × cm	30 × 30 × 1.99	30 × 30 × 2.00	30 × 30 × 1.97	30 × 30 × 2.16
**Porosity**	%	25.9	24.8	24.3	22.2
**Absolute Permeability**	mD	1706	1554	1783	2510
**S_wi_**	fraction	0.11	0.14	0.07	0.11
**Oil Viscosity**	cP (mPas)	478	487	433	467
**Injection Rate: Q = 0.05 mL/min**					
**Water Breakthrough**	PV inj.	0.09	0.24	0.17	0.55
**Total Recovery**	% OOIP	31	56	67	72
**dP @ end**	mbar	21	87	45	96

**Table 2:** Pore network parameters.

Parameter	Unit	Value
Network size	-	50 × 25 × 1
Coordination number	-	4
Pore size distribution model	µm	$\bar{r}$ = 18, σ = 9
Minimum inscribed radius	µm	10
Maximum inscribed radius	µm	50
Permeability	mD	2069
Distortion factor	-	0.3
Average pore length	µm	333
Pore half angles	-	30, 30, 30
Wettability	-	Water wet
Water/oil contact angle	Degree	0
Interfacial tension	N/m	0.0004
S_wi_	-	0
Injection rate	m^3^/s	1 × 10^−12^
Capillary No. (waterflood)	-	3.28 × 10^−6^
Oil viscosity	cP (mPas)	466

**Figure A1:**
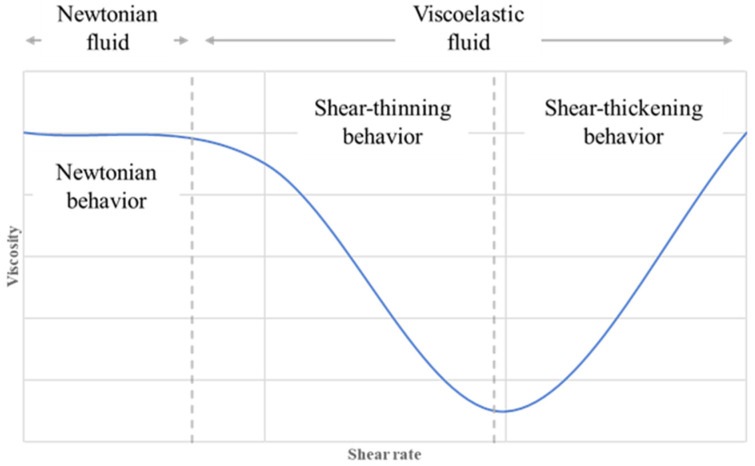
Schematic demonstration of both shear thinning and thickening fluid behaviour in porous media.

## Appendix A. Model Description

### Appendix A.1. The Dynamic Imbibition Pore Network Model for EOR

The pore network model applied was first developed by Li et al. (2017) [30] as a dynamic imbibition model, which includes both piston-like displacement and snap-off events that may occur simultaneously. Both viscous and capillary forces are considered, which is more appropriate for studying enhanced oil recovery methods at the pore scale. In this model, a switch parameter, λ, is used, as defined by Equation (A1). It is a rate-dependent parameter expressing the ratio of the capillary force to the total (capillary + viscous) forces acting in a local pore filling event. This parameter simulates the local competition between capillary and viscous force to determine primary pore filling mechanisms, which can be piston-like displacement, snap-off/film swelling, or a combination of these two mechanisms. The limits of λ are—when λ = 1 only capillary forces are present, and if λ = 0, only viscous forces operate.

(A1) $$\lambda =\frac{P_{c}}{P_{c}+\Delta P}$$

In more water-wet conditions, water occupies the corners of pores as a (“thick”) film and provides connected pathways. Thereby, during the imbibition processes, both piston-like displacement and snap-off may occur, while, for a drainage process, only piston-like displacement can happen. In this model, piston-like displacement can proceed if upstream adjacent bulk water is available and pressure drop from the water-filled end to oil-filled end of the pore is larger than entry capillary pressure ($\Delta P\geq \;-P_{c}$). During snap-off, water will accumulate in water films in the corners and, if it swells sufficiently, the fluid-fluid interface becomes unstable and snap-off then occurs. The decision on volume of water which contributes to either piston-like displacement or film swelling is determined by the switch parameter, λ.

After defining the filling mechanisms and fluid configurations inside each pore, the bond conductance can be calculated. Li et al. (2017) [30] describe the calculation procedure based on fluid configurations inside each bond. Based on the bond conductivity, the water flow rate in each bond can be described by Equation (A2) without bulk menisci and by Equation (A3) with menisci. In these equations, *q* is the bond flow rate, *g* is the bond conductivity, *P_c_* is the entry capillary pressure, and *P_i_* and *P_j_* are the pressures of nodes at two ends of the corresponding bond.

(A2) $$q=g\;\left(P_{i}-P_{j}\right)$$

(A3) $$q=g\;\left(P_{i}-P_{j}+P_{c}\right)$$

By considering mass conservation law for each node ($\sum_{i=1}^{z}q_{i}=0$), a set of linear equations of the form of **A****∙****p** **= b** arise and are solved numerically, where **A** is a matrix of a (known) coefficient and includes conductance of bonds, **b** is the ‘right hand side’ (known) vector and includes boundary conditions as well as entry capillary pressures, and **p** is the (unknown) vector of pressures at each node. By solving this system of linear equations, the pressure at each node is obtained and, consequently, the flow rate will be updated for each bond.

To solve the system of the equation, boundary conditions are at a constant pressure at the inlet and outlet. However, similar to lab experiments, constant global pressure drop along the sample should be so that it maintains a predefined constant injection rate ($Q_{inj}$). To do so, an iterative approach is used. Based on Aker’s method [33], the governing equation can be simplified as $Q=a\left(\Delta P+\;{\bar{P}}_{c,\;entry}\right)=a\;\Delta P+b$. To obtain parameters a and b, an iterative approach is used as follows.

- Choose arbitrary $P_{in}$ and $P_{out}$ ($\Delta P_{init}=P_{in}-P_{out}$) and calculate $Q_{inlet}^{I}$
- Choose Pin = Pout and calculate $Q_{inlet}^{II}$
- Based on the calculated $Q_{inlet}^{I}$ and $Q_{inlet}^{II}$ in the above two steps, calculate using Equations (A4) and (A5).
  (A4) $$b=\;Q_{inlet}^{II}$$
  (A5) $$a=\frac{Q_{inlet}^{I}-Q_{inlet}^{II}}{\Delta P_{init}}$$
- Update pressure at the inlet using the following equation (A6).
  (A6) $$P_{in}=\frac{Q_{inj}-b}{a}+P_{out}$$
- Repeat the above four steps until a satisfactory accuracy between a predefined injection rate ($Q_{inj}$) and $Q_{inlet}^{I}$ is obtained.

After calculating pressures for each node, bond flow rates are then updated. Bond flow rates are used to calculate the minimum time steps and the new local λ values to update water volume in each bond, through the appropriate (λ dependent) mechanism. The minimum time step is chosen so that, in each time step, water can only fill, at most, one complete pore.

### Appendix A.2. Modifying the Dynamic Imbibition Model to Include Polymer Displacements

As noted already, polymer solutions are non-Newtonian and may demonstrate one of the following behaviors in terms of flow.

(a) Newtonian behavior: Polymer viscosity depends on polymer concentration but is independent of a shear rate (flow rate).
(b) Shear thinning behavior: Polymer viscosity decreases as the shear rate increases.
(c) Shear thickening: polymer viscosity increases as the shear rate increases.
(d) Combination of all or some of the previously mentioned behaviors.

All the above can be modelled using Equation (A7), which is a general form of viscosity behaviour as a function of shear rate, which includes Newtonian, shear thinning, and shear thickening behaviour and combinations of these, by an appropriate choice of parameters.

(A7) $$\mu =\;\underset{shear\;viscosity}{\underbrace{\mu_{\infty }+\left(\mu_{0}-\mu_{\infty }\right){\left[1+{\left(\lambda_{p}\dot{\gamma \;}\right)}^{2}\right]}^{\left(n-1\right)/2}}}\;+\;\underset{Extensional\;viscosity}{\underbrace{\mu_{max}\left[1-exp\left(-{\left(\tau_{r}\dot{\gamma \;}\right)}^{n_{2}-1}\right)\right]}}$$

where $\mu_{\infty }$ is the high shear Newtonian plateau viscosity and is considered here as water viscosity (1 cP), while $\mu_{0}$ is the upper low shear Newtonian plateau, which is the maximum polymer viscosity at low shear rates. $\mu_{max}$ is the maximum polymer viscosity at high shear rates when polymer solution demonstrates shear thickening properties. $\lambda_{p}$ and $\tau_{r}$ define the onset of shear thinning and shear thickening behaviours, respectively. n and $n_{2}$ define the slope of shear thinning and shear thickening parts, respectively. Possible rheology models arising from this formulation are shown schematically in Figure A1. All the parameters in Equation A are functions of a polymer concentration [16]. However, among all, only $\mu_{0}$ and $\mu_{max}$ are considered as a linear function of polymer concentration and the rest are constant predefined values. $\dot{\gamma }$ is the local shear rate (in a pore) and, in this work, the shear rate is linearly related to local velocity ($\dot{\gamma }=\alpha \;v$). α is a parameter to relate the shear rate to velocity, which is a function of microscopic properties of porous media and polymer properties. Zamani et al. (2017) [34] have described in more detail how to relate the shear rate to local velocity.

To include the polymer rheology in the two-phase dynamic imbibition model, we first needed to calculate the local polymer concentration, which is modelled by incorporating a transport equation for the polymer in the aqueous phase in the network model. A dimensionless polymer concentration, C, is defined where 0 ≤ C ≤ 1, with C = 1 being the injected polymer concentration. The polymer is injected at the inlet in one of following ways: (a) continuous injection starting from time = 0 of network simulation, (b) polymer injection after some period of water injection, and (c) polymer injection for some period, which is followed by water injection. In all cases, concentrations are updated using mass conservation law based on concentration/flow field. We assume that the polymer can flow through both bulk and water films.
